# A five-year prospective study of spinal radiographic progression and its predictors in men and women with ankylosing spondylitis

**DOI:** 10.1186/s13075-018-1665-1

**Published:** 2018-08-03

**Authors:** Anna Deminger, Eva Klingberg, Mats Geijer, Jan Göthlin, Martin Hedberg, Eva Rehnberg, Hans Carlsten, Lennart T. Jacobsson, Helena Forsblad-d’Elia

**Affiliations:** 1 Department of Rheumatology and Inflammation Research, Sahlgrenska Academy at University of Gothenburg, Box 480, 405 30 Gothenburg, Sweden; 2 Department of Radiology, Skåne University Hospital, 221 85 Lund, Sweden; 3 Faculty of Medicine, Lund University, Box 117, 221 00 Lund, Sweden; 4 Department of Radiology, Sahlgrenska University Hospital, Mölndal, 431 80 Mölndal, Sweden; 5 Section of Rheumatology, Södra Älvsborg Hospital, 501 82 Borås, Sweden; 6 Section of Rheumatology, Alingsås Hospital, 441 33 Alingsås, Sweden; 7 Department of Public Health and Clinical Medicine, Rheumatology, 901 87 Umeå University, Umeå, Sweden

**Keywords:** Ankylosing spondylitis, Outcomes research, Treatment, Inflammation, Longitudinal study, Radiography

## Abstract

**Background:**

Knowledge about predictors of new spinal bone formation in patients with ankylosing spondylitis (AS) is limited. AS-related spinal alterations are more common in men; however, knowledge of whether predictors differ between sexes is lacking. Our objectives were to study spinal radiographic progression in patients with AS and investigate predictors of progression overall and by sex.

**Methods:**

Swedish patients with AS, age (mean ± SD) 50 ± 13 years, were included in a longitudinal study. At baseline and at 5-year follow up, spinal radiographs were graded according to the modified Stoke Ankylosing Spondylitis Spine Score (mSASSS). Predictors were assessed by questionnaires, spinal mobility tests and blood samples.

**Results:**

Of 204 patients included, 166 (81%) were re-examined and 54% were men. Men had significantly higher mean mSASSS at baseline and higher mean increase in mSASSS than women (1.9 ± 2.8 vs. 1.2 ± 3.3; *p* = 0.005) More men than women developed new syndesmophytes (30% vs. 12%; *p* = 0.007). Multivariate logistic regression analyses with progression ≥ 2 mSASSS units over 5 years or development of new syndesmophytes as the dependent variable showed that presence of baseline AS-related spinal radiographic alterations and obesity (OR 3.78, 95% CI 1.3 to 11.2) were independent predictors of spinal radiographic progression in both sexes. High C-reactive protein (CRP) was a significant predictor in men, with only a trend seen in women. Smoking predicted progression in men whereas high Bath Ankylosing Spondylitis Metrology Index (BASMI) and exposure to bisphosphonates during follow up (OR 4.78, 95% CI 1.1 to 20.1) predicted progression in women.

**Conclusion:**

This first report on sex-specific predictors of spinal radiographic progression shows that predictors may partly differ between the sexes. New predictors identified were obesity in both sexes and exposure to bisphosphonates in women. Among previously known predictors, baseline AS-related spinal radiographic alterations predicted radiographic progression in both sexes, high CRP was a predictor in men (with a trend in women) and smoking was a predictor only in men.

**Trial registration:**

ClinicalTrials.gov, NCT00858819. Registered on 9 March 2009. Last updated 28 May 2015.

## Background

Ankylosing spondylitis (AS) is a chronic, inflammatory disease mainly affecting the sacroiliac joints and the spine, where it is characterized by pathological new bone formation and development of syndesmophytes. The new bone formation and spinal inflammation can lead to spinal stiffness and loss of mobility [1]. The ratio of men to women with AS is estimated to be 2–3 to 1 [2].

Conventional x-ray is still considered the gold standard for assessing chronic spinal alterations in AS, with the modified Stoke Ankylosing Spondylitis Spine Score (mSASSS) considered as the most valid method for quantifying these changes [3]. AS-related spinal alterations evaluated by different methods have been shown to be more severe in men [4–6] and longitudinal studies have shown faster radiographic progression in men [6, 7].

There is still limited knowledge on predictors of spinal radiographic progression in AS. The strongest predictor is the presence of syndesmophytes at baseline [5, 8–10]. Higher disease activity measured by the Ankylosing Spondylitis Disease Activity Score (ASDAS) has been associated with more spinal radiographic progression in AS and early axial spondyloarthritis (SpA) [11, 12]. Inflammation measured by erythrocyte sedimentation rate (ESR) or C-reactive protein (CRP) and smoking has been shown to predict radiographic progression in early SpA [10]. Previous studies have largely been in men and knowledge about what predicts radiographic progression in women is scarce.

The objectives of this longitudinal study were to assess spinal radiographic progression in patients with AS and to investigate predictors for progression overall and by sex.

## Methods

### Patients

The patients were recruited at baseline in 2009 from the rheumatology clinics at Sahlgrenska University Hospital in Gothenburg and the hospitals at Borås and Alingsås, Sweden [13]. The inclusion criterion was AS according to the modified New York criteria [14]. Exclusion criteria were psoriasis, inflammatory bowel disease, dementia, pregnancy and difficulties in understanding the Swedish language. A total of 204 patients completed the baseline protocol and were invited to participate in the 5-year follow up. Written informed consent was obtained from the participants and approval by the regional ethics committee in Gothenburg was obtained both at baseline and at 5-year follow up.

### Physical examination and questionnaires

The same questionnaires and physical examinations, including the Bath AS Metrology Index (BASMI) [15] were applied at baseline and at the 5-year follow up. Patients were examined by one physician at baseline (EK) and one physician at follow up (AD). Questionnaires included medical history, medication, occupation and smoking and the Bath AS Disease Activity Index (BASDAI), the Bath AS Functional Index (BASFI) and the Bath AS Patient Global score (BAS-G) [16–18]. The ASDAS based on CRP (ASDAS_CRP) was calculated [19]. Type of occupation was divided into “blue-collar” work, generally involving manual labor and physical tasks, and “white-collar” work, usually requiring less physical activity and more formal education [20]. Body mass index (BMI) was grouped in categories: 1 = normal (BMI 18.5 to 24.9 kg/m^2^), 2 = overweight (BMI 25.0 to 29.9), 3 = obese (BMI ≥ 30) [21]. Data about non-steroidal anti-inflammatory drug (NSAID) consumption during follow up was collected according to the Assessment of SpondyloArthritis international Society (ASAS) recommendations [22]. All patients were invited to undergo transthoracic echocardiography at baseline as previously described [23].

### Radiography

Conventional radiographs of the spine were obtained at baseline and at the 5-year follow up and scored according to the mSASSS. With mSASSS, each anterior corner of the cervical spine (from the lower corner of vertebra C2 to the upper corner of T1) and the lumbar spine (from the lower corner of T12 to the upper corner of S1) on lateral radiographs is evaluated with a score between 0 and 3 (0 = no abnormality, 1 = erosion, sclerosis or squaring, 2 = syndesmophyte, 3 = bridging syndesmophyte), with the total score ranging from 0 to 72 [24]. All radiographs were scored simultaneously by the same musculoskeletal radiologist (MG) blinded to the clinical data but with known chronological order. At baseline, 0.3% of the vertebral corners (VC) were not assessable and 0% were not assessable at follow up. Missing VC was handled according to Ramiro et al. [7] where missing baseline VC was replaced by the score from the next observation with the mean progression sum of the segment subtracted from that score. Missing follow-up VC was replaced by the score of the previous observation with the mean progression score of the segment added to that score. No patient had missing VC at both baseline and follow up. Definite radiographic progression was defined either as an increase in mSASSS over 5 years by ≥ 2 points [25] or as development of a new syndesmophyte, defined as mSASSS of at least 2 at a vertebral level with a score of 0 or 1 at baseline [5].

### Laboratory tests

Blood samples were analyzed by standard laboratory techniques. The time-averaged ESR and CRP for the last 5 years before follow up were obtained from the medical records, and calculated using the first recorded test for each year unless the patient had a recorded infection at that time point, in which case the ESR/CRP was replaced by the subsequent test.

### Statistics

Statistical analyses were performed using IBM SPSS Statistics 22 (IBM, Armonk, NY, USA). Descriptive statistics are presented as means with standard deviations (SD) and frequencies with percentages. To compare measurements in men and women the *t* test for normally distributed data or the Mann-Whitney U test for not normally distributed data were used for continuous data, and the chi^2^ test or Fisher’s exact test, when appropriate, was used for categorical data. The Wilcoxon signed rank test was used to compare mSASSS at baseline and at follow up. Radiographs of 40 randomly selected patients were re-scored by the same reader for calculations of reliability data. Intra-reader agreement for status scores and change scores was evaluated with an intraclass correlation coefficient (ICC) two-way mixed-effect model, with single measurement and absolute agreement. Values < 0.5 indicate poor agreement, 0.5–0.75 moderate agreement, 0.75–0.90 good agreement and > 0.90 excellent agreement [26]. The smallest detectable change (SDC), the progression reliably detected above the measurement error, was calculated as proposed by Bruynesteyn et al. [27].

Univariate and multivariate (backward method) logistic regression analyses were performed to find predictors for progression of ≥ 2 mSASSS units over 5 years and the development of new syndesmophytes. Variables with *p* values ≤ 0.2 in the univariate analyses were entered into the multivariate logistic regression model for the total group. Because of fewer observations in the subgroups, a *p* value < 0.1 was used for sex-stratified analyses. Variables were entered manually into the multivariate model and if there was multicollinearity (BASMI and BASFI), the variable with the lowest *p* value in the univariate analyses was kept in the model. The same principle was used for highly correlated variables: age and duration of symptoms and BASMI and lateral spinal flexion (as lateral spinal flexion is a part of BASMI) but for CRP, time-averaged CRP, ESR and time-averaged ESR the baseline variable was prioritized. For mSASSS and BASMI and mSASSS and lateral flexion (highly correlated in the total group and in men), the mSASSS was chosen. Interactions between the main effect variables from the multiple logistic analyses were tested, and if a significant interaction (*p* ≤ 0.05) was identified, the estimates for progression in the subgroups were analyzed.. Goodness of fit was assessed with the Hosmer-Lemeshow test.

To consider covariates that affect receiving bisphosphonates or tumor necrosis factor inhibitor (TNFi), propensity scores (PS) for the probabilities of being exposed to bisphosphonates, being exposed to TNFi or being treated with TNFi for ≥ 2 years during follow up, respectively, were calculated. Variables included in the PS were sex, HLA-B27, baseline CRP, BASDAI, mSASSS, smoking pack-years, BMI categories, use of NSAID, symptom duration and age of onset of symptoms. The PS and the treatment variable were used as covariates in standard binary logistic regression analyses with either definition of progression as the dependent variable. In order to evaluate any interaction between NSAID and TNFi-exposure, three categorical groups were formed: high NSAID (NSAID index ≥ 50) and TNFi+/−, low NSAID (NSAID index < 50) and TNFi+/− and no NSAID and TNFi+/−. Each group was used as a covariate either in univariate or together with the PS for exposure to TNFi in multivariate logistic regression analyses for radiographic progression, as aforementioned. All tests were two-tailed and *p* ≤ 0.05 was considered statistically significant.

## Results

### Patients

**Fig. 1:**
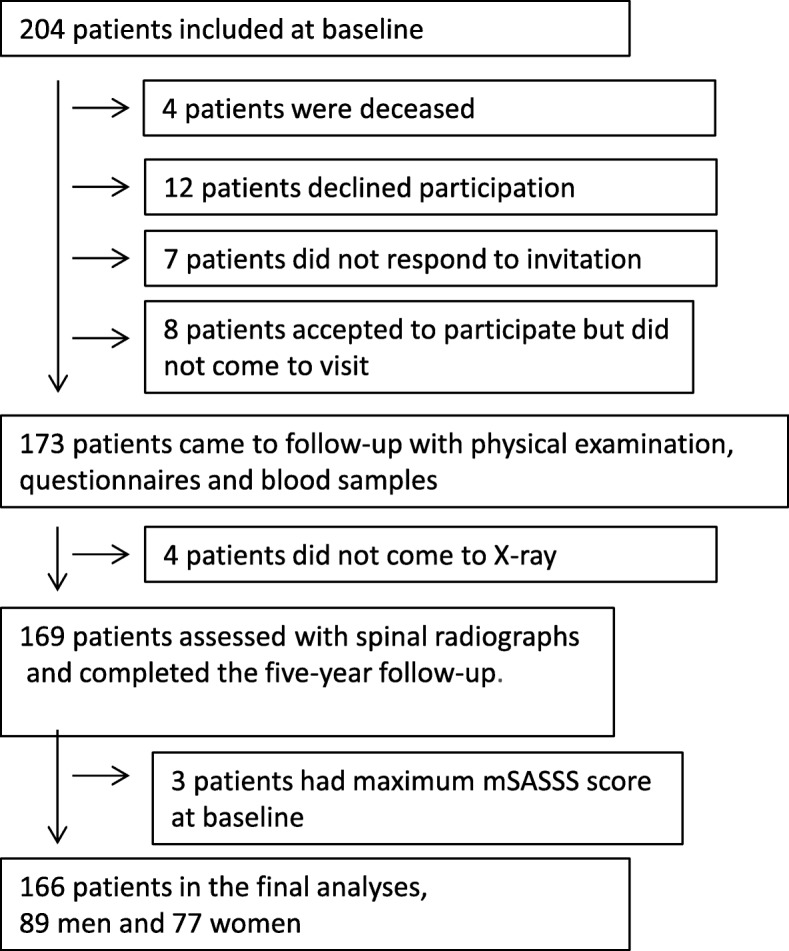
Flow chart shows participation from baseline to the 5-year follow up. mSASSS, modified Stoke Ankylosing Spondylitis Spine Score

Of the 204 patients included at baseline, 169 (83%) completed all examinations at the 5-year follow up: 4 patients died during follow up. Three men with maximum mSASSS at baseline were excluded from the analyses, resulting in 166 (81%) completers, including 89 men (54%) and 77 women (46%) (Fig. 1). The 166 completers did not differ in baseline age or mSASSS compared to the non-completers; the non-completers included patients coming to follow up with baseline mSASSS = 72 and those not coming to follow up (50 ± 13 vs. 50 ± 14 years). There was a trend towards more men in the non-completers vs. the completers (71% vs. 54%, *p* = 0.078), and there was no significant difference when analyzing only patients who were still alive at follow-up (*p* = 0.19).

Characteristics and medication use in the total group and a comparison between men and women at baseline are shown in Table 1. There were some significant differences between men and women; more men were HLA-B27-positive, men had lower ESR, higher time-averaged CRP during follow up, and a trend toward lower disease activity measured by the BASDAI. Men had higher mean (SD) mSASSS at baseline than women (20.3 (21.9) vs. 6.4 (9.6), *p* < 0.001) and 57% of the men had syndesmophytes compared with 33% of the women (*p* = 0.002*)*. At baseline, 78% of the patients reported using NSAIDs and 20% had treatment with TNFi, with no significant differences between sexes. Bisphosphonates were used by a smaller proportion of men than women (1% vs. 8%, *p* = 0.050).

**Table 1:** Baseline characteristics and medication at baseline and during follow up in 166 patients with ankylosing spondylitis

	Total group	Men (*n* = 89)	Women (*n* = 77)	*p* value
Demographic variables				
Age, years	50 (13)	49 (12)	51 (13)	0.45
Current smokers	17 (10)	8 (9)	9 (12)	0.75
Ever-smokers	72 (43)	41 (46)	31 (40)	0.55
BMI category: normal/overweight/obese	85 (51)/	41 (46)/	44 (57)/	0.36
	56 (34)/	33 (37)/	23 (30)/	
	25 (15)	15 (17)	10 (13)	
Blue-collar worker^a^	40 (33)	21 (33)	19 (33)	1.00
Time between x-rays, months	66 (3)	65 (3)	67 (1)	0.003
Disease-related variables				
Duration of symptoms, years	24 (13)	23 (13)	24 (13)	0.58
History of anterior uveitis	85 (51)	50 (56)	35 (46)	0.22
History of peripheral arthritis	95 (57)	47 (53)	48 (62)	0.28
History of coxitis	13 (8)	7 (8)	6 (8)	1.00
BASMI, score	3.0 (1.5)	3.2 (1.8)	2.9 (1.2)	0.61
BASFI, score	2.5 (2.0)	2.3 (1.9)	2.7 (2.1)	0.25
BASDAI, score	3.4 (2.1)	3.1 (2.1)	3.7 (2.0)	0.056
ASDAS_CRP, score	2.1 (0.9)	2.1 (0.9)	2.1 (0.8)	0.53
CRP, mg/L	5.4 (8.5)	6.5 (10.5)	4.2 (5.1)	0.26
Time-averaged CRP during follow up, mg/L	5.8 (5.9)	6.5 (6.4)	4.9 (5.0)	0.043
ESR, mm/h	14.2 (11.2)	12.7 (11.5)	15.9 (10.6)	0.003
Time-averaged ESR during follow-up, mm/h	12.2 (8.4)	10.9 (7.8)	13.8 (8.9)	0.009
HLA-B27 positive	143 (86)	82 (92)	61 (79)	0.030
Aortic insufficiency^b^	25 (16)	13 (16)	12 (17)	0.98
mSASSS, score	13.9 (18.6)	20.3 (21.9)	6.4 (9.6)	< 0.001
Presence of syndesmophyte	76 (46)	51 (57)	25 (33)	0.002
Medications				
Patients on NSAIDs baseline	129 (78)	66 (74)	63 (82)	0.32
NSAID index during follow up, 0–100	34 (38)	39 (39)	29 (33)	0.31
Patients on TNFi at baseline	33 (20)	22 (25)	11 (14)	0.14
Patients exposed to TNFi during follow up	49 (30)	30 (34)	19 (25)	0.27
Patients on bisphosphonate at baseline	7 (4)	1 (1)	6 (8)	0.050
Patients exposed to bisphosphonate during follow-up	30 (18)	11 (12)	19 (25)	0.064

Values are means (SD) or numbers of patients (%)

*ASDAS_CRP* Ankylosing Spondylitis Disease Activity Score_C-reactive protein, *BASDAI* Bath Ankylosing Spondylitis Disease Activity Index, *BASFI* Bath Ankylosing Spondylitis Functional Index, *BASMI* Bath Ankylosing Spondylitis Metrology Index, *BMI* body mass index, *CRP* C-reactive protein, *ESR* erythrocyte sedimentation rate, *HLA-B27* human leukocyte antigen B27, *mSASSS* modified Stoke Ankylosing Spondylitis Spine Score, *NSAID* non-steroidal anti-inflammatory drug, *TNFi* tumor necrosis factor inhibitor

^a^*n* = 120 for total group, 63 men and 57 women

^b^*n* = 153 for total group, 83 men and 70 women

### Spinal radiographic progression

**Fig. 2:**
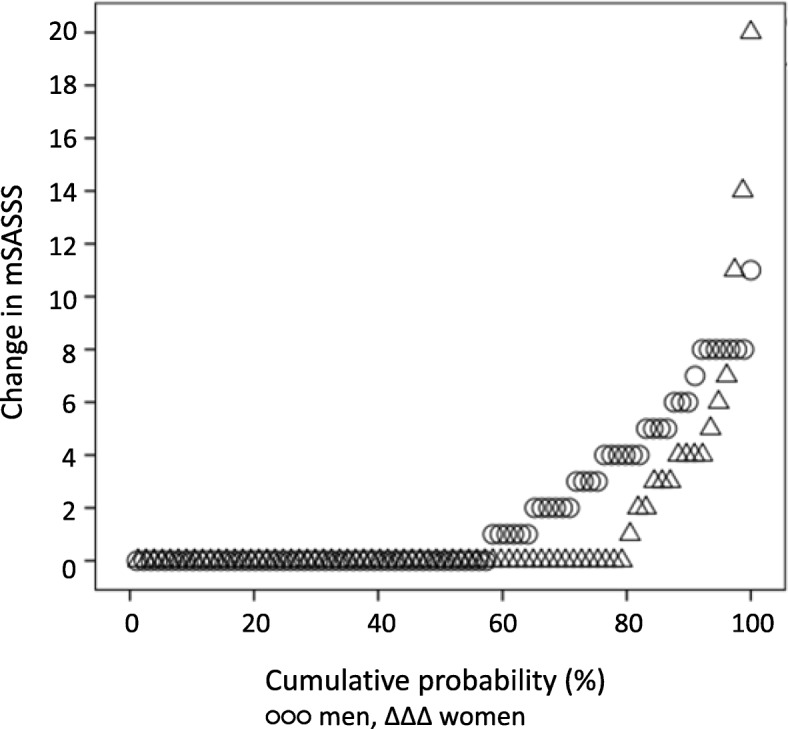
Cumulative probability for the change in modified Stoke Ankylosing Spondylitis Spine Score (mSASSS) from baseline to follow up in 89 men and 77 women with ankylosing spondylitis

In the total group, mSASSS progressed from mean (SD) 13.9 (18.6) units to 15.4 (19.6) units. The mean progression was 1.6 (3.3) mSASSS units over 5 years (*p* < 0.001), with more progression in men compared to women (1.9 (2.8) vs 1.2 (3.3), *p* = 0.005) (Fig. 2). The mSASSS ranged from 0 to 70 in men at baseline and from 0 to 72 at follow up. In women the range was 0–46 at baseline and 0–57 at follow up. Five of the completers had baseline mSASSS > 65. One of these patients reached a maximum mSASSS of 72 at follow up. This patient fulfilled both definitions of progression. The ICC for status scores were 0.98 (95% CI 0.96 to 0.99) for both baseline and follow-up scores and 0.62 (95% CI 0.36 to 0.78) for change scores. The SDC was 2.65.

Progression ≥ 2 mSASSS units over 5 years was seen in 47 patients (28%), more frequently in men than in women, with 32 men (36%) vs. 15 women (20%) (*p* = 0.029). The development of new syndesmophytes was seen in 36 patients (22%), with 27 men (30%) and 9 women (12%) (*p* = 0.007).

### Predictors of progression defined as increase of ≥ 2 mSASSS units or new syndesmophyte development over 5 years in univariate analyses (Tables 2 and 3)

Whole study population: several predictors were the same for both definitions of progression. Demographic variables that predicted progression were male sex, older age, and being overweight or obese. Disease-BRS81665 related variables and medication that predicted progression were history of anterior uveitis, high BASMI, reduced lateral spinal flexion, presence of syndesmophytes at baseline and exposure to bisphosphonates during follow up. Inflammation measured by CRP predicted progression of ≥ 2 mSASSS units over 5 years.

**Table 2:** Univariate logistic regression analyses for progression of ≥ 2 mSASSS units over 5 years

	Total group, *n* = 166			Men, n = 89			Women, n = 77		
	OR	95% CI	*p* value	OR	95% CI	*p* value	OR	95% CI	*p* value
Demographic variables									
Male sex	2.32	1.14 to 4.72	**0.020**	NA			NA		
Age, years	1.03	1.01 to 1.06	**0.021**	1.03	1.00 to 1.07	0.078	1.05	1.00 to 1.10	0.075
BMI: overweight (reference, normal)	2.40	1.09 to 5.26	**0.029**	2.62	0.95 to 7.21	0.062	1.76	0.47 to 6.54	0.40
obese (reference, normal)	5.06	1.93 to 13.24	**0.001**	5.33	1.50 to 19.0	**0.010**	4.22	0.91 to 19.51	0.065
Ever-smoker	1.74	0.88 to 3.44	0.11	3.53	1.42 to 8.77	**0.007**	0.47	0.14 to 1.65	0.24
Current smoker	1.44	0.50 to 4.14	0.50	3.33	0.74 to 15.00	**0.12**	0.48	0.06 to 4.18	0.51
Blue-collar work^a^	1.31	0.55 to 3.12	0.55	1.00	0.31 to 3.19	1.00	1.91	0.50 to 7.29	0.35
Time between x-rays, months	0.92	0.81 to 1.05	0.21	0.96	0.83 to 1.11	0.57	1.15	0.66 to 2.00	0.63
Disease-related variables									
Duration of symptoms, years	1.02	0.99 to 1.04	0.24	1.01	0.98 to 1.05	0.57	1.03	0.99 to 1.08	0.17
HLA-B27 positive	1.50	0.52 to 4.30	0.45	1.44	0.26 to 7.90	0.67	1.06	0.26 to 4.33	0.93
History of coxitis	0.74	0.20 to 2.83	0.66	0.27	0.03 to 2.39	0.24	2.23	0.37 to 13.51	0.38
History of uveitis	2.33	1.16 to 4.71	**0.018**	2.28	0.92 to 5.66	0.076	2.08	0.66 to 6.56	0.21
BASMI, score	1.65	1.29 to 2.10	**< 0.001**	1.43	1.10 to 1.86	**0.008**	2.58	1.42 to 4.69	**0.002**
Lateral spinal flexion, cm	0.89	0.84 to 0.96	**0.001**	0.91	0.84 to 0.98	**0.014**	0.83	0.71 to 0.96	**0.012**
BASFI, score	1.15	0.97 to 1.36	0.10	1.13	0.90 to 1.42	0.28	1.27	0.96 to 1.67	0.089
BASDAI, score	0.98	0.83 to 1.15	0.80	0.93	0.76 to 1.15	0.52	1.17	0.87 to 1.56	0.30
ASDAS_CRP, score	1.14	0.78 to 1.68	0.51	1.12	0.70 to 1.80	0.64	1.24	0.63 to 2.46	0.54
CRP, mg/L	1.06	1.02 to 1.11	**0.008**	1.05	1.00 to 1.10	0.051	1.09	0.99 to 1.21	0.083
Time-averaged CRP, mg/L	1.08	1.02 to 1.14	**0.010**	1.09	1.01 to 1.17	**0.035**	1.05	0.95 to 1.16	0.36
ESR, mm/h	1.02	0.99 to 1.05	0.19	1.02	0.98 to 1.06	0.39	1.04	0.99 to 1.09	0.10
Time-averaged ESR, mm/h	1.03	0.99 to 1.07	0.11	1.05	0.99 to 1.11	0.097	1.04	0.98 to 1.10	0.21
Syndesmophytes at baseline	6.46	2.98 to 14.03	**< 0.001**	5.55	1.98 to 15.54	**0.001**	6.27	1.85 to 21.24	**0.003**
mSASSS baseline, units	1.04	1.02 to 1.05	**< 0.001**	1.02	1.00 to 1.04	**0.046**	1.11	1.04 to 1.19	**0.001**
Aortic insufficiency^b^	1.20	0.48 to 3.03	0.70	1.20	0.35 to 4.06	0.77	1.28	0.30 to 5.46	0.74
Medication									
NSAID index, 0–100	1.01	1.00 to 1.01	0.26	1.00	0.99 to 1.02	0.52	1.00	0.99 to 1.02	0.62
Exposure to TNFi	1.17	0.56 to 2.43	0.67	0.84	0.33 to 2.12	0.71	1.71	0.50 to 5.85	0.39
Exposure to bisphosphonates	2.29	1.01 to 5.21	**0.047**	1.57	0.44 to 5.63	0.49	5.30	1.59 to 17.69	**0.007**

ORs reflect a change in one unit for continuous variables. All variables are baseline values except for time-averaged variables and medications. Significant *p* values are shown in bold typeface

*ASDAS_CRP* Ankylosing Spondylitis Disease Activity Score_C-reactive protein, *BASDAI* Bath Ankylosing Spondylitis Disease Activity Index, *BASFI* Bath Ankylosing Spondylitis Functional Index, *BASMI* Bath Ankylosing Spondylitis Metrology Index, *BMI* body mass index, *CRP* C-reactive protein, *ESR* erythrocyte sedimentation rate, *HLA-B27* Human leukocyte antigen B27, *mSASSS* modified Stoke Ankylosing Spondylitis Spine Score, *NSAID* non-steroidal anti-inflammatory drug, *TNFi* tumor necrosis factor inhibitor

*n* = 120 for total group, *n* = 63 for men, *n* = 57 for women

^‡^*n* = 153 for total group, *n* = 83 for men and *n* = 70 for women

**Table 3:** Univariate logistic regression analyses for development of new syndesmophytes over 5 years

	Total group, n = 166			Men, n = 89			Women, n = 77		
	OR	95% CI	*p* value	OR	95% CI	*p* value	OR	95% CI	*p* value
Demographic variables									
Male sex	3.29	1.44 to 7.54	**0.005**	NA			NA		
Age, years	1.05	1.02 to 1.09	**0.003**	1.06	1.02 to 1.10	**0.007**	1.06	0.99 to 1.13	0.082
BMI: overweight (reference, normal)	2.46	1.04 to 5.85	**0.042**	3.16	1.08 to 9.22	**0.036**	0.95	0.16 to 5.63	0.96
obese (reference, normal)	4.49	1.62 to 12.44	**0.004**	4.25	1.16 to 15.60	**0.029**	4.29	0.78 to 23.43	0.093
Ever-smoker	1.22	0.58 to 2.56	0.60	1.73	0.70 to 4.31	0.24	0.38	0.07 to 1.99	0.25
Current smoker	2.16	0.74 to 6.32	0.16	4.47	0.99 to 20.29	0.052	0.94	0.10 to 8.51	0.95
Blue-collar work^a^	1.18	0.45 to 3.10	0.74	1.47	0.44 to 4.87	0.53	0.78	0.14 to 4.43	0.78
Time between x-rays	0.82	0.71 to 0.94	**0.004**	0.88	0.75 to 1.02	0.083	0.75	0.36 to 1.56	0.44
Disease-related variables									
Duration of symptoms, years	1.02	0.99 to 1.05	0.13	1.02	0.99 to 1.06	0.26	1.04	0.98 to 1.09	0.18
HLA-B27 positive	1.00	0.34 to 2.90	1.00	0.55	0.12 to 2.65	0.46	0.91	0.17 to 4.86	0.91
History of coxitis	1.09	0.28 to 4.19	0.90	0.36	0.04 to 3.14	0.35	4.57	0.71 to 29.61	0.11
History of uveitis	2.26	1.04 to 4.91	**0.039**	1.88	0.73 to 4.81	0.19	2.69	0.62 to 11.66	0.19
BASMI, score	1.57	1.22 to 2.01	**< 0.001**	1.29	1.00 to 1.68	0.053	4.39	**1.77 to 10.84**	**0.001**
Lateral spinal flexion, cm	0.89	0.83 to 0.96	**0.002**	0.92	0.85 to 1.00	**0.036**	0.71	0.55 to 0.91	**0.006**
BASFI, score	1.14	0.95 to 1.36	0.16	1.11	0.88 to 1.40	0.40	1.35	0.97 to 1.87	0.072
BASDAI, score	1.01	0.85 to 1.21	0.91	1.01	0.82 to 1.26	0.90	1.14	0.81 to 1.61	0.45
ASDAS_CRP	1.23	0.81 to 1.86	0.34	1.19	0.73 to 1.95	0.48	1.44	0.63 to 3.32	0.39
CRP, mg/L	1.02	0.99 to 1.06	0.24	1.02	0.98 to 1.06	0.46	1.01	0.89 to 1.15	0.86
Time-averaged CRP, mg/L	1.06	1.00 to 1.12	0.060	1.05	0.98 to 1.13	0.15	1.03	0.91 to 1.16	0.65
ESR, mm/h	0.99	0.95 to 1.02	0.43	1.00	0.96 to 1.04	0.83	0.99	0.92 to 1.06	0.69
Time-averaged ESR, mm/h	0.99	0.94 to 1.03	0.60	1.00	0.94 to 1.06	0.94	1.00	0.93 to 1.09	0.93
Syndesmophytes at baseline	5.98	2.52 to 14.17	**< 0.001**	5.01	1.68 to 14.92	**0.004**	5.16	1.17 to 22.74	**0.030**
Aortic insufficiency^b^	1.53	0.58 to 4.04	0.40	1.11	0.31 to 4.02	0.87	2.89	0.61 to 13.69	0.18
Medication									
NSAID index, 0–100	1.01	1.00 to 1.02	0.11	1.01	1.00 to 1.02	0.11	1.00	0.97 to 1.02	0.72
Exposure to TNFi	1.07	0.48 to 2.38	0.88	0.59	0.22 to 1.62	0.31	2.83	0.67 to 11.86	0.16
Exposure to bisphosphonates	2.57	1.09 to 6.08	**0.031**	2.12	0.59 to 7.67	0.25	8.46	1.87 to 38.38	**0.006**

ORs reflect a change in one unit for continuous variables. All variables are baseline values except for time-averaged variables and medications. Significant *p* values are shown in bold typeface

*ASDAS_CRP* Ankylosing Spondylitis Disease Activity Score_C-reactive protein, *BASDAI* Bath Ankylosing Spondylitis Disease Activity Index, *BASFI* Bath Ankylosing Spondylitis Functional Index, *BASMI* Bath Ankylosing Spondylitis Metrology Index, *BMI* body mass index, *CRP* C-reactive protein, *ESR* erythrocyte sedimentation rate, *HLA-B27* Human leukocyte antigen B27, *mSASSS* modified Stoke Ankylosing Spondylitis Spine Score, *NSAID* non-steroidal anti-inflammatory drug, *TNFi* tumor necrosis factor inhibitor

^*a*^*n* = 120 for total group, *n* = 63 for men, *n* = 57 for women

^b^*n* = 153 for total group*n* = 83 for men and *n* = 70 for women

Sex-stratified analyses: obesity predicted progression in men according to both definitions. Ever-smoking and high CRP during follow up predicted progression of ≥ 2 mSASSS units over 5 years. Older age and being overweight predicted development of new syndesmophytes. In women exposure to bisphosphonates predicted progression according to both definitions. Shared predictors in both men and women were disease-related variables such as high BASMI, reduced lateral spinal flexion and presence of syndesmophytes at baseline (according to both definitions for progression).

### Predictors of progression defined as increase of ≥ 2 mSASSS units or new syndesmophyte development over 5 years in multivariate analyses (Figs. 3 and 4)

Whole study population: in multivariate analyses exposure to bisphosphonates during follow up was associated with progression according to both definitions. Obesity, high baseline CRP and male sex predicted progression of ≥ 2 mSASSS units over 5 years. Baseline syndesmophytes and older age predicted development of new syndesmophytes. For development of new syndesmophytes, the point estimates in the model were similar irrespective of whether baseline CRP or time-averaged CRP were included in the model.

**Fig. 3:**
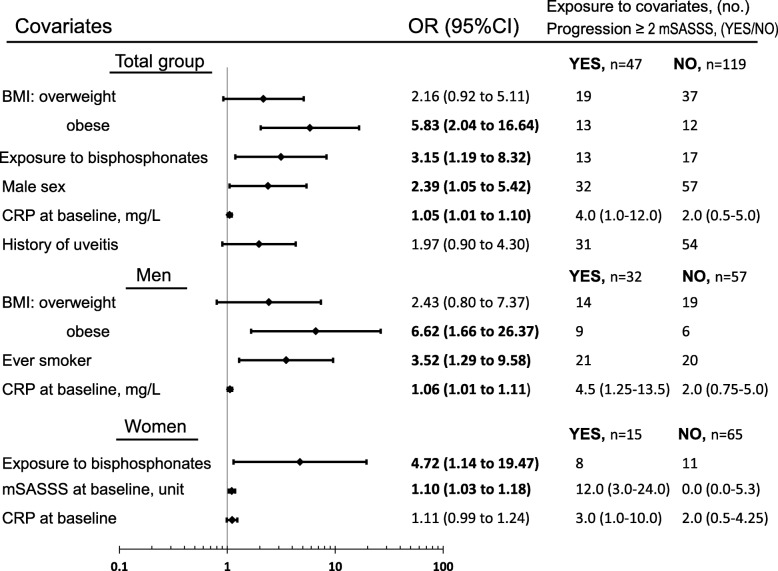
Multivariate logistic regression analyses of predictors of progression of ≥ 2 modified Stoke Ankylosing Spondylitis Spine Score (mSASSS) units over 5 years. ORs reflect a change in one unit for continuous variables. Normal body mass index (BMI) = reference. Exposure data are number of patients exposed or median (interquartile range). Significant ORs are shown in bold typeface. Variables included for total group: BMI categories, exposure to bisphosphonates, sex, baseline C-reactive protein (CRP), history of uveitis, age, ever-smoker, baseline mSASSS and Bath Ankylosing Spondylitis Functional Index (BASFI), for men: BMI categories, ever-smoker, baseline CRP, age, history of uveitis and baseline mSASSS, for women: exposure to bisphosphonates, baseline mSASSS, baseline CRP, age, BMI categories and BASMI. CI, confidence interval; OR, odds ratio

**Fig. 4:**
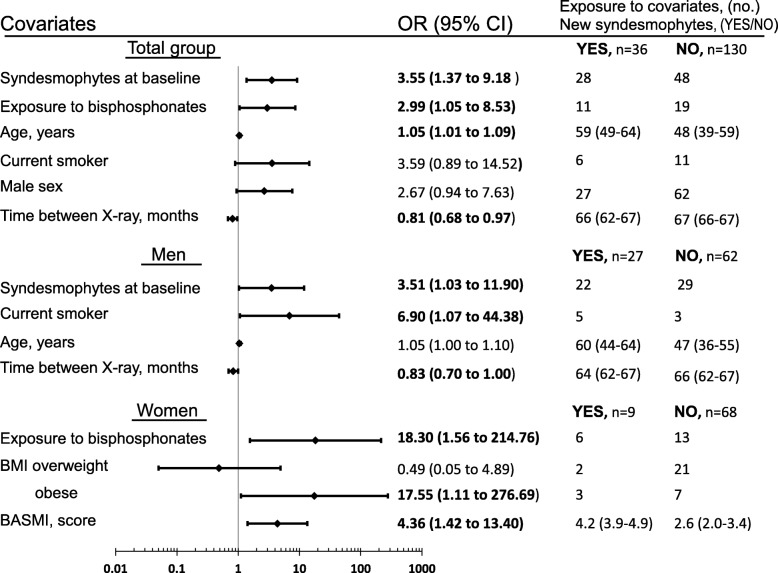
Multivariate logistic regression analyses for predictors of development of new syndesmophytes. ORs reflect a change in one unit for continuous variables. Normal body mass index (BMI) = reference. Exposure data are number of patients exposed or median (interquartile range). Significant ORs are shown in bold typeface. Variables included for total group: baseline syndesmophytes, exposure to bisphosphonates, age, current smoker, sex, time between x-rays, BMI categories, history of uveitis, baseline C-reactive protein (CRP) or time-averaged CRP, non-steroidal anti-inflammatory drug (NSAID)-index and Bath Ankylosing Spondylitis Metrology Index (BASMI), for men: baseline syndesmophytes, current smoker, age, time between x-rays, BMI categories and lateral spinal flexion, for women: exposure to bisphosphonates, BMI categories, BASMI, age and baseline syndesmophytes. CI, confidence interval; OR, odds ratio

Sex-stratified analyses: in men, ever-smoking, obesity and high baseline CRP predicted progression defined as ≥2 mSASSS units over 5 years. Current smoking and presence of baseline syndesmophytes predicted development of new syndesmophytes. In women, exposure to bisphosphonates was independently associated with progression according to both definitions. Baseline mSASSS predicted progression defined as ≥ 2 mSASSS over 5 years whereas there was only a trend for baseline CRP. Obesity and high BASMI predicted development of new syndesmophytes. BASMI also remained significant if the model was corrected for baseline mSASSS instead of baseline syndesmophytes.

A significant interaction was identified for age and exposure to bisphosphonates on the development of new syndesmophytes. When stratifying by median age (≥ 50 vs. < 50 years), the estimates for exposure to bisphosphonates for the respective group was not significant (OR 1.34 95% CI 0.49 to 3.66 vs. OR 4.56 95% CI 0.72 to 28.95).

### Effect of treatment with bisphosphonates in PS-adjusted analyses in the whole study population

Of the 30 patients exposed to bisphosphonates during follow up, 7 patients had used bisphosphonates at baseline. The remaining 23 patients started the medication soon after inclusion, at mean (SD) 7 (0.5) months. The PS for the probability of being exposed to bisphosphonates during follow up was calculated as described in “Methods”. In the logistic regression model with the PS included, exposure to bisphosphonates was still a significant predictor for development of new syndesmophytes (OR 2.96, 95% CI 1.02 to 8.62), but was not statistically significant for progression of ≥ 2 mSASSS units over 5 years (Table 4).

**Table 4:** Effect of bisphosphonates and TNFi, respectively, on spinal radiographic progression in propensity score-adjusted logistic regression analyses

	Progression ≥ 2 mSASSS units		New syndesmophytes	
	OR (95% CI)	*p* value	OR (95% CI)	*p* value
Exposure to bisphosphonates	2.47 (0.89 to 6.86)	0.083	2.96 (1.02 to 8.62)	**0.046**
Propensity score, 0–1	0.32 (0.03 to 3.65)	0.36	0.41 (0.03 to 5.59)	0.51
Exposure to TNFi	0.91 (0.40 to 2.08)	0.83	1.05 (0.43 to 2.52)	0.92
Propensity score, 0–1	6.26 (0.78 to 49.89)	0.084	1.18 (0.12 to 11.30)	0.89
TNFi for ≥ 2 years	0.86 (0.35 to 2.09)	0.73	0.81 (0.30 to 2.21)	0.68
Propensity score, 0–1	3.78 (0.41 to 34.593)	0.24	0.69 (0.06 to 8.46)	0.77

Significant *p* value is shown in bold typeface

*mSASSS* modified Stoke Ankylosing Spondylitis Spinal Score, *TNFi* tumor necrosis factor inhibitor

### Effect of treatment with TNFi in PS-adjusted analyses in the whole study population

Exposure to TNFi during follow up was not significant (*p* value >0.2) in the univariate analyses of association with radiographic progression (Tables 2 and 3) and was thus not included in the multivariate models. To further study associations between TNFi and spinal radiographic progression, the PS for exposure to TNFi or use of TNFi for ≥ 2 years during follow up was calculated as described in “Methods”. In the logistic regression model with the PS included, exposure to TNFi (*n* = 49) or use of TNFi for ≥ 2 years during follow up (*n* = 38) was still not significantly associated with radiographic progression (Table 4).

To analyze if concomitant use of NSAID and TNFi could have an impact on spinal progression, the patients were grouped according to NSAID and TNFi use during follow up as described in “Methods”. There was no significant relationship between dose of NSAID and concomitant use of TNFi in these regression analyses (Table 5). However, only 10 patients had a combination of high-dose NSAID and exposure to TNFi.

**Table 5:** Effect of TNFi on spinal radiographic progression according to NSAID use, with and without propensity score adjustment

	Univariate OR (95% CI)	*p* value	OR (95% CI) with PS^a^	*p* value
Progression ≥ 2 mSASSS units				
TNFi vs. no TNFi and no NSAID	3.33 (0.46 to 24.05)	0.23	3.06 (0.27 to 34.70)	0.37
TNFi vs. no TNFi and low NSAID	1.24 (0.46 to 3.38)	0.67	1.16 (0.40 to 3.40)	0.79
TNFi vs. no TNFi and high NSAID	0.74 (0.17 to 3.31)	0.70	0.48 (0.09 to 2.48)	0.38
New syndesmophytes				
TNFi vs. no TNFi and no NSAID	1.25 (0.14 to 10.94)	0.84	2.51 (0.17 to 38.18)	0.51
TNFi vs. no TNFi and low NSAID	1.26 (0.42 to 3.81)	0.67	1.44 (0.44 to 4.70)	0.55
TNFi vs. no TNFi and high NSAID	1.04 (0.23 to 4.69)	0.96	0.84 (0.17 to 4.21)	0.83

*BASDAI* Bath Ankylosing Spondylitis Disease Activity Index, *BMI* body mass index, *CRP* C-reactive protein, *HLA-B27* Human leucocyte antigen B27, *mSASSS* modified Stoke Ankylosing Spondylitis Spine Score, *NSAID* non-steroidal anti-inflammatory drug, *PS* propensity score, *TNFi* tumor necrosis factor inhibitor

^a^Variables included in PS are sex, HLA-B27, baseline CRP, BASDAI, mSASSS, smoking pack-years, BMI category, symptom duration and age of onset of symptoms

## Discussion

In the present study, we investigated spinal radiographic progression and its predictors in men and women separately and demonstrated a higher occurrence and development of syndesmophytes in men. Shared predictors of progression in both sexes were the presence of baseline AS-related spinal radiographic alterations and obesity, whereas exposure to smoking may be a more important predictor in men and exposure to bisphosphonates may be more important in women.

The higher occurrence of AS-related spinal alterations in men has also been shown in other studies [4, 6]. Ramiro et al. also identified faster progression in men [7]. Since AS-related spinal radiological changes are more common in men, predictors should be studied separately for men and women in the same setting. To our knowledge, this has not been done previously. In one prior study in women with AS, older age, longer disease duration, severe sacroiliitis, elevated CRP and baseline syndesmophytes were predictors of the development of new syndesmophytes in the lumbar spine over 2 years in univariate analyses. Multivariate analysis was not done due to the small sample size [28]. Several longitudinal studies on mixed gender cohorts have reported, similar to the current study, preexisting syndesmophytes as a predictor in both long-standing AS and early SpA [5, 9, 10, 25]. Elevated CRP or ESR, smoking and high disease activity over time measured by ASDAS_CRP have been independently associated with spinal radiographic progression [11, 29, 30]. Of these, high CRP and smoking were predictors of radiographic progression in men in the current Swedish cohort. The non-significant result for high CRP as a predictor in women might be due to the small sample size whereas the fact that the univariate ORs estimate indicates a negative effect of smoking on radiographic progression in men and a positive effect in women, may imply a real difference between sexes. Based on previous observations a larger sample size would, however, have been required to have the statistical power to detect any significant difference between sexes in the effect of smoking [29]. Intriguingly, exposure to bisphosphonates during follow up was found to be a predictor of spinal radiographic progression in women. Bisphosphonates have been studied as disease-modifying drugs in AS [31–33]. One of these trials explored spinal radiographic progression and observed no difference in mSASSS progression over 2 years in patients randomized to alendronate compared with placebo [33]. However, in that study, few patients on alendronate were women and not all patients were radiographed. Our results are based on few observations and should be interpreted with caution, and our finding needs confirmation in a larger study with more women. Neither NSAID nor TNFi treatment was associated with spinal radiographic progression but the study was not designed to evaluate treatment effects. Few patients were treated with TNFi, with substantial variation in treatment duration and different starting points. The PS for TNFi treatment was also based on baseline variables as variables at other starting points were not available.

Obesity was found to be a predictor of spinal radiographic progression in both sexes. A previous cross-sectional study identified higher BMI in patients with syndesmophytes, which is in line with our findings [34]. whereas a recently published longitudinal study found no association with being overweight or obese and radiographic progression [35]. Obesity is associated with higher bone mineral density (BMD) in the general population, this being attributed to greater mechanical loading and hormones. Also, adipokines secreted by adipose tissue have an effect on BMD, although has not yet been fully elucidated [36]. Adipokines also have an effect on immune functions and inflammatory processes in the body [37] but the knowledge of the role of adipokines in AS is limited and conflicting. Two cross-sectional studies found elevated leptin to be associated with the presence of syndesmophytes [34, 38], whereas one longitudinal study demonstrated a protective effect of leptin against spinal radiographic progression [39] and another found elevated visfatin at baseline to predict spinal progression [40]. Mechanical loading has been shown to result in new bone formation in mice, an effect that is not yet proven in humans [41]. Ramiro et al. used a physically demanding occupation as a proxy for “life time mechanical stress” on the spine and found that blue-collar work amplified the effect of inflammation on radiographic progression [42]. Doran et al., on the other hand, found no association between AS-related radiological changes and occupational activity level in a retrospective study of patients with AS [43]. In the current study there was no association between occupation at baseline and radiographic progression.

High BASMI independently predicted the development of new syndesmophytes in women. The BASMI has to our knowledge not been studied as a predictor of spinal radiographic progression before, but could be an interesting predictor as it is clinically more feasible than, for example, radiographic examinations. The BASMI is correlated with the mSASSS but the variables are not interchangeable [44]. Impaired spinal mobility is more influenced by inflammation in early AS and by spinal radiographic changes later in the disease [45].We do not believe that high BASMI causes the progression but rather that it follows from a high mSASSS.

Progression of mSASSS varied considerably between patients. However, the mean progression of 1.6 mSASSS units over 5 years in the current study was lower than in previous reports on patients with long-standing AS. For instance, previous studies in three different AS cohorts with 100%, 20% and 0% of the patients treated with TNFi showed a progression of mean 1.3 mSASSS units per 2 years [46], 2.0 mSASSS units per 2 years [7, 11] and 1.3 mSASSS units per year [9], respectively. The reason for the modest mSASSS increase in the current study is not obvious, especially since the radiographic alterations at baseline were similar to those in the OASIS cohort [7, 11]. Possibly, it could be explained by differences in the selection of patients. Another factor could be the smaller proportion of men in this Swedish cohort, but not even the progression in men of 1.9 mSASSS units over 5 years was on a level with the previously reported progression rates. Osteo-proliferative changes in the present study were scored with knowledge of the serial order of acquisition of the radiographs, and reading in serial order is more sensitive to change, hence, progression should not have been underestimated [47].

The patients in the current study were recruited from rheumatology clinics and may have more severe disease. However, 70 patients (42%) among the completers had been referred to a general practitioner during follow up due to inactive disease and the percentage of patients using TNFi at baseline was similar to that in Swedish patients with AS in a nationwide register-based report from the same year (20% in our cohort vs 17% in all Swedish patients with AS) [48]. At baseline, the patients with AS were somewhat older in the current study than the patients that declined participation, as reported previously [49]. At follow up, the completers did not differ in age or mSASSS compared with non-completers, but there was a trend toward there being more women among the completers. Even if we cannot exclude the possibility that there has been some selection bias towards older patients and women in this study, we believe that our patients are representative of patients with AS in our region.

In order to decrease radiographic progression in the spine we propose the supporting of weight loss in obese patients, counseling on the hazardous effect of smoking and treatment of active inflammation. In addition, further and larger studies on the effect of bisphosphonates on radiographic progression in the spine are needed.

One limitation of the present study is the relatively small number of patients in the subgroups, especially of women among whom few have spinal radiographic progression, and in particular the development of new syndesmophytes. This reduces the statistical power as displayed by the large confidence intervals in the results for women. Thus, the results for the subgroups, and in particular for women, need to be interpreted with caution and confirmed in a larger cohort with more women. Another limitation is the use of only one reader of the radiographs. Strengths of this study are the long follow-up time, the prospective longitudinal design with well-characterized patients and many variables identified and analyzed that have potential association with osteo-proliferation. It is also the first study reporting sex-specific predictors of spinal radiographic progression in AS.

## Conclusion

Over 5 years, men had greater spinal radiographic progression than women and predictors of progression differed partly between sexes. New predictors identified were obesity in both sexes and exposure to bisphosphonates and impairment of spinal mobility in women. Among previously known predictors, baseline spinal radiographic alterations was a predictor in both sexes, high CRP was a predictor in men, with a trend in women too, whereas smoking predicted progression in men.

## Abbreviations

AS: Ankylosing spondylitis
ASAS: Assessment of SpondyloArthritis international Society
ASDAS: Ankylosing Spondylitis Disease Activity Score
ASDAS_CRP: Ankylosing Spondylitis Disease Activity Score based on C-reactive protein
BASDAI: Bath Ankylosing Spondylitis Disease Activity Index
BASFI: Bath Ankylosing Spondylitis Functional Index
BAS-G: Bath Ankylosing Spondylitis Patient Global Score
BASMI: Bath Ankylosing Spondylitis Metrology Index
BMI: Body mass index
CI: Confidence interval
CRP: C-reactive protein
ESR: Erythrocyte sedimentation rate
HLA-B27: Human leukocyte antigen B27
ICC: Intraclass correlation coefficient
mSASSS: modified Stoke Ankylosing Spondylitis Spine Score
NSAID: Non-steroid anti-inflammatory drug
OR: Odds ratio
PS: Propensity score
SD: Standard deviation
SDC: Smallest detectable change
SpA: Spondyloarthritis
TNFi: Tumor necrosis factor inhibitor
